# Geometric morphological parameters from cardiac magnetic resonance imaging in assessment of chronic pulmonary thromboembolism, a prospective pilot study

**DOI:** 10.1186/s41747-026-00811-1

**Published:** 2026-09-21

**Authors:** Jie Du, Yanlin Guo, Hanchi Yu, Anqi Liu, Linfeng Xi, Shuai Zhang, Yunxia Zhang, Wanmu Xie, Yong Cheng, Min Liu

**Affiliations:** 1 Chinese Academy of Medical Sciences & Peking Union Medical College, Beijing, 100730 China; 2 Department of Radiology, China-Japan Friendship Hospital, Beijing, 100029 China; 3 Peking University China-Japan Friendship School of Clinical Medicine, Beijing, 100191 China; 4 China-Japan Friendship Hospital, Department of Pulmonary and Critical Care Medicine, Beijing, 100029 China; 5 School of Information Science and Technology, Beijing University of Chemical Technology, Beijing, 100029 China

**Keywords:** Fractal dimension, Magnetic resonance imaging, Pulmonary embolism, Perimeter-area ratio, Ventricular remodeling

## Abstract

**Objective:**

The role of geometric-morphological parameters derived from cardiac magnetic resonance imaging (CMR) in assessing and stratifying chronic pulmonary thromboembolism (CPTE) remains unclear.

**Material and methods:**

We prospectively enrolled 53 CPTE patients (16 with chronic thromboembolic pulmonary disease (CTEPD), 37 with chronic thromboembolic pulmonary hypertension (CTEPH)) and 53 age- and sex-matched healthy controls. All patients underwent CMR and right heart catheterization within 24 h. Biventricular fractal dimension (FD) and perimeter-area ratio (PAR) were quantified on short-axis cine images at end-diastole and end-systole across basal, mid, and apical levels. Group comparisons, ROC analysis, and Spearman correlations were performed. Incremental diagnostic value was assessed using multivariable logistic regression, net reclassification improvement, and integrated discrimination improvement.

**Results:**

Right ventricular (RV) FD was significantly higher and RVPAR lower in CTEPH, most pronounced at the apical level, both in end-diastole and end-systole (*p* < 0.05). Apical RVFD and RVPAR robustly differentiated CPTE from controls (AUC = 0.911–0.961) and CTEPH from CTEPD (AUC = 0.819–0.895). Both parameters provided significant incremental diagnostic value over RVEF. In univariable analysis, RVFD correlated positively with afterload indices and negatively with perfusion/output metrics; RVPAR showed inverse associations (all *p* < 0.05). Multivariable linear regression showed that RVPAR retained weak independent negative associations with mean pulmonary arterial pressure and pulmonary vascular resistance.

**Conclusion:**

CMR-derived apical RVFD and RVPAR reflect early, load-dependent ventricular remodeling in CPTE and demonstrated potential as complementary non-invasive imaging markers for severity assessment and risk stratification, highlighting the apex as the most sensitive site of RV adaptation.

**Key Points:**

- **Question** *The role of CMR-derived fractal dimension and perimeter-area ratio in assessing right ventricular remodeling and differentiating CTEPH from CTEPD remains unclear.*
- **Findings** *Apical RVFD and RVPAR differentiated CPTE from controls (AUC 0.911-0.961) and CTEPH from CTEPD (AUC 0.819-0.895), correlated with PVR and mPAP, and provided incremental diagnostic value over RVEF.*
- **Relevance statement** *This pilot study suggests that cine-derived fractal dimension and perimeter-area ratio can characterize right ventricular remodeling, correlate with invasive hemodynamics, and provide incremental diagnostic value over conventional functional indices.*

**Graphical Abstract:**

**Figure:**
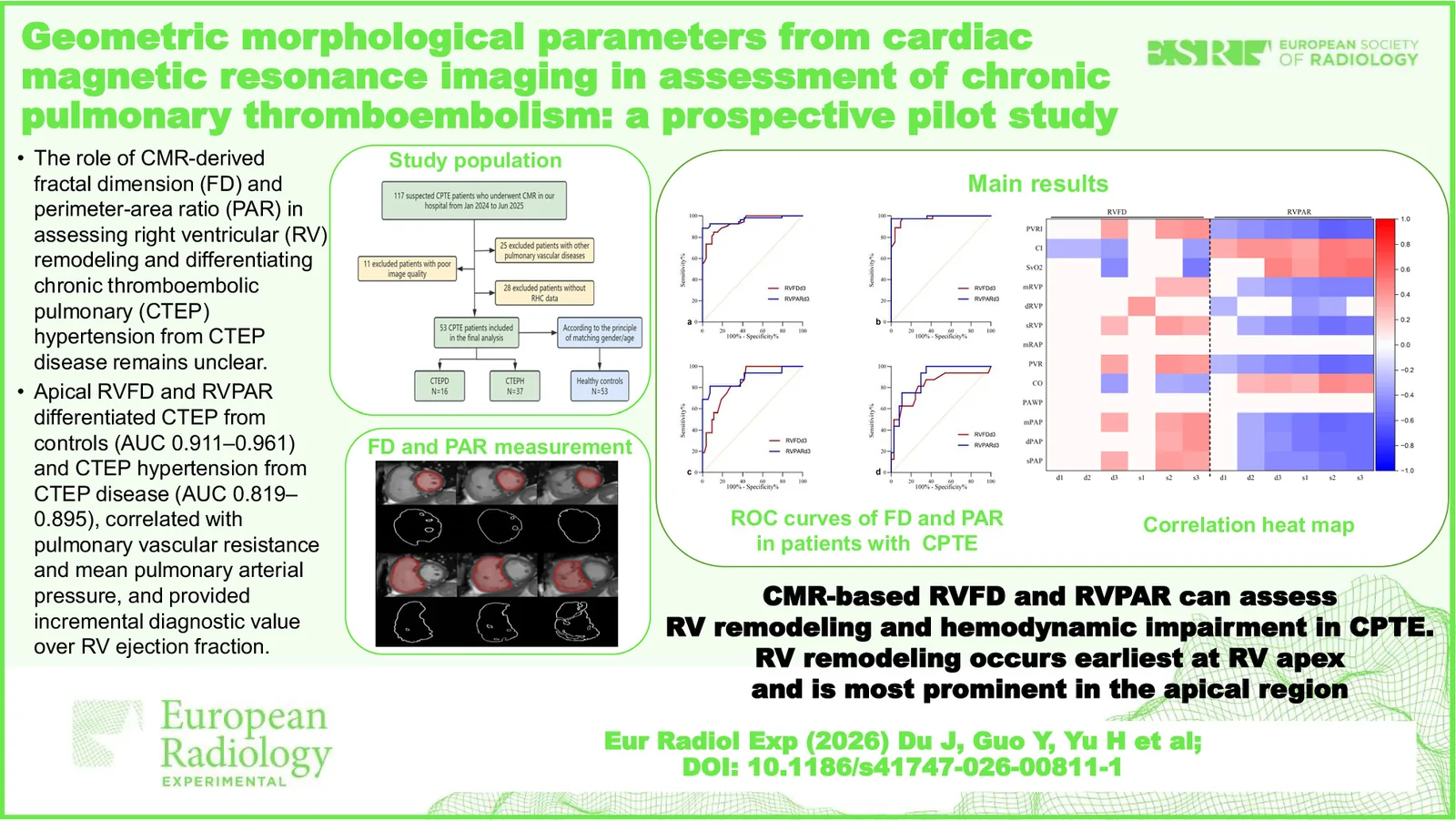

## Introduction

Chronic pulmonary thromboembolism (CPTE) is characterized by the persistence of organized thrombi in the pulmonary arteries for more than three months following an acute pulmonary thromboembolism (APTE), despite adequate anticoagulation [1]. Depending on the presence of resting pulmonary hypertension, CPTE is further classified into chronic thromboembolic pulmonary disease (CTEPD) and chronic thromboembolic pulmonary hypertension (CTEPH) [1]. Although CTEPH occurs in approximately 2.7% of APTE survivors [2], it represents the most severe long-term complication, potentially progressing to right heart failure and death. Due to its nonspecific symptoms and the reliance on invasive right heart catheterization (RHC) for definitive diagnosis, CTEPH is often diagnosed at an advanced stage.

Cardiac magnetic resonance imaging (CMR) is a non-invasive, radiation-free modality that provides accurate quantification of ventricular volumes and function [3]. However, conventional CMR parameters may lack sensitivity in detecting early myocardial alterations during compensated remodeling. Morphological analysis provides a novel approach for quantifying pathological cardiac remodeling. Fractal dimension (FD) and perimeter-area ratio (PAR) are metrics that quantify structural complexity in two- or three-dimensional space. FD has been widely applied to assess complexity in various organ systems, including the lungs [4, 5], vasculature [6, 7], heart [8, 9], bone [10], and brain [11]. In pulmonary hypertension (PH), FD has demonstrated high sensitivity and translational potential in evaluating right ventricular (RV) trabecular complexity [12], pulmonary vascular architecture [13, 14] and lung perfusion heterogeneity [15]. While PAR has been utilized in fields such as melanoma detection [16], its role in CPTE remains unexplored. Given the distinct cardiac remodeling patterns in CTEPH *versus* CTEPD, we hypothesized that FD and PAR could capture progressive, phenotype-specific ventricular changes across the spectrum from health to CTEPD and CTEPH. This study aimed to systematically measure biventricular FD and PAR from CMR in patients with CTEPD, CTEPH, and healthy controls, evaluate their correlations with hemodynamic parameters and incremental clinical value beyond conventional CMR functional indices, and explore their potential as non-invasive imaging biomarkers for the diagnosis and risk stratification of CPTE.

## Materials and methods

### Study population

**Fig. 1:**
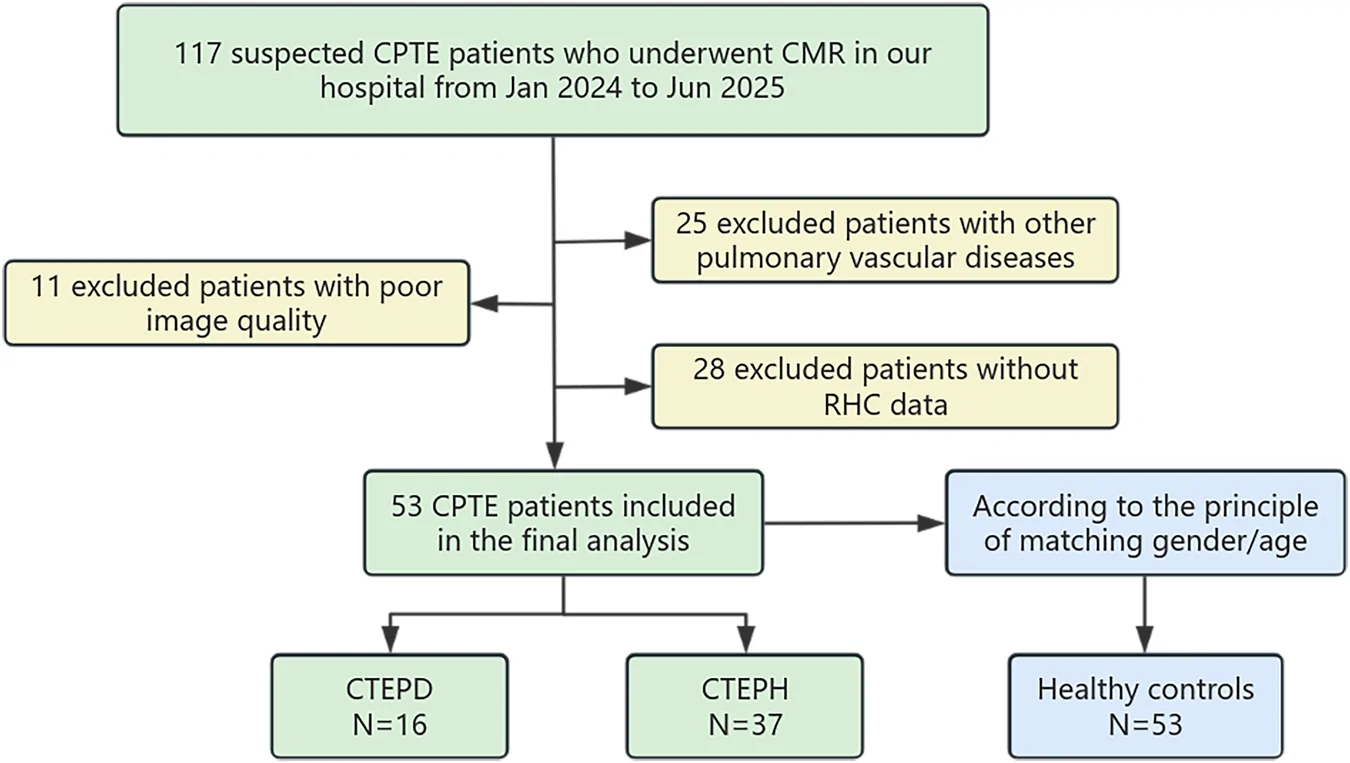
Flowchart of the study. CPTE, Chronic pulmonary thromboembolism; CMR, Cardiac magnetic resonance imaging; RHC, Right heart catheterization; CTEPD, Chronic thromboembolic pulmonary disease; CTEPH, Chronic thromboembolic pulmonary hypertension

This prospective, single-center pilot study was approved by the ethics committees of our hospital and registered at ClinicalTrials.gov. All participants provided written informed consent prior to CMR. Between Jan 2024 and Jun 2025, 53 patients with CPTE were enrolled at our hospital, including 37 with CTEPH and 16 with CTEPD. Diagnosis was established according to the 2022 ESC/ERS consensus guidelines [1], which require confirmation of CPTE by computed tomography pulmonary angiography (CTPA) or ventilation/perfusion (V/Q) scintigraphy after at least 3 months of adequate anticoagulation. All CPTE patients had received stable anticoagulation therapy for at least 3 months, including 48 on rivaroxaban and 5 on warfarin, and all underwent CMR and RHC within 24 h. Meanwhile, 53 sex- and age-matched healthy volunteers were recruited to undergo non-contrast CMR. Exclusion criteria were as follows: (1) poor CMR image quality due to respiratory artifacts or severe arrhythmias; (2) an interval exceeding 24 hours between CMR and RHC; (3) a final diagnosis of other pulmonary vascular diseases, including acute pulmonary embolism, Takayasu arteritis, Behçet’s disease, fibrosing mediastinitis, pulmonary artery sarcoma, or other forms of pulmonary hypertension; (4) incomplete RHC data. The study flowchart is presented in Fig. 1.

### CMR acquisition and functional analysis

CMR was performed on a 1.5-Tesla scanner (MAGNETOM Aera, Siemens Healthcare, Erlangen, Germany) using an 18-channel phased-array coil. Imaging included retrospective ECG-gated, breath-held, balanced steady-state free precession sequences without contrast. Standard short-axis stacks covered both ventricles from base to apex, supplemented by four-chamber long-axis views. The typical acquisition parameters included repetition time (TR) = 34.5 ms, flip angle (FA) = 50–60°, slice thickness = 6 mm, in-plane spatial resolution 1.8 × 1.8 mm^2^, temporal resolution 40 ms, and 25 reconstructed cardiac phases. Shimming and center frequency adjustments were performed as needed to minimize off-resonance artifacts.

Cine images in Digital Imaging and Communications in Medicine (DICOM) format were transferred to the SyngoVia workstation (Siemens Healthcare Sector, Forchheim, Germany). Left ventricular (LV) endocardial and epicardial contours at end-diastole (ED) and end-systole (ES) were automatically segmented; RV contours were manually delineated by two radiologists with 5 years of cardiovascular imaging experience. Biventricular functional parameters were subsequently derived.

### Morphological parameter analysis

**Fig. 2:**
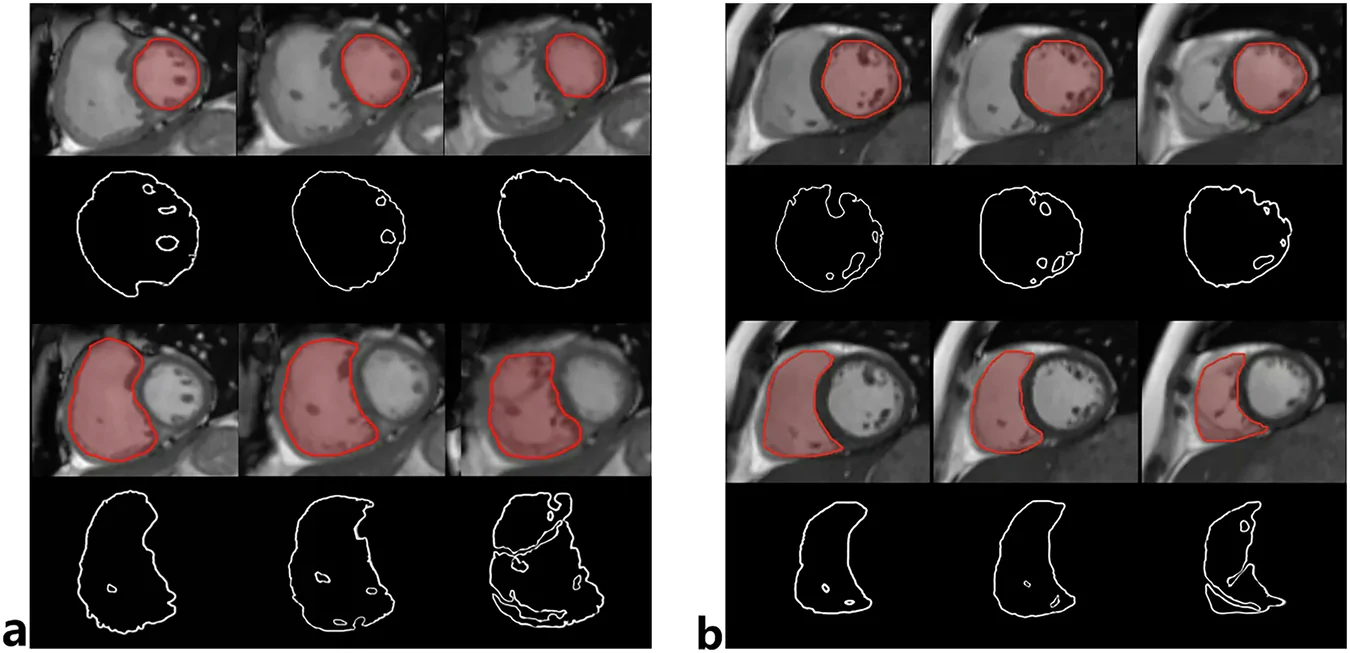
FD and PAR measurement on cine. **a** FD and PAR measurement on the short-axis cine in a patient with CTEPH (male, age = 51 years old). RVFDd1 = 1.248, RVFDd2 = 1.255, RVFDd3 = 1.257; RVPARd1 = 0.138, RVPARd2 = 0.129, RVPARd3 = 0.132; LVFDd1 = 1.207, LVFDd2 = 1.213, LVFDd3 = 1.217; LVPARd1 = 0.243, LVPARd2 = 0.320, LVPARd3 = 0.251; **b** FD and PAR measurement on the short-axis cine in a patient with CTEPD (male, age = 51 years old). RVFDd1 = 1.251, RVFDd2 = 1.247, RVFDd3 = 1.250; RVPARd1 = 0.155, RVPARd2 = 0.162, RVPARd3 = 0.198; LVFDd1 = 1.219, LVFDd2 = 1.207, LVFDd3 = 1.199; LVPARd1 = 0.302, LVPARd2 = 0.238, LVPARd3 = 0.221. RVFD, Right ventricular fractal dimension; RVPAR, Right ventricular perimeter-area ratio; LVFD, Left ventricular fractal dimension; LVPAR, Left ventricular perimeter-area ratio; d1, End-diastolic basal level; d2, End-diastolic papillary muscle level; d3, End-diastolic apical level; s1, End-systolic basal level; s2, End-systolic papillary muscle level; s3, End-systolic apical level

As shown in Fig. 2, three standard short-axis slices were selected from cine images at ED and ES for both ventricles: the apical level (below the tips of the papillary muscles), the papillary muscle level (at the mid-level of the papillary muscles), and the basal level (at the level of the mitral valve annulus). Cine images in the DICOM format were imported into Python 3.12, and then ventricular endocardial borders were manually segmented by two cardiovascular radiologists with 5 years’ experience, who were blinded to group allocation, along the endocardial-blood pool interface, excluding papillary muscles and chordae tendineae. Code (https://github.com/chengyong/cmrifd) leveraged the scikit-image library to extract endocardial and trabecular contours, followed by regional property analysis and parameter calculation.

### Biventricular fractal dimension (FD)

FD was computed using the box-counting method to characterize the complexity and self-similarity of myocardial contour boundaries. The box size s decreased logarithmically; for each s, the number of non-zero pixels, N(s), was counted in the downsampled binary mask. A linear relationship was observed between log *N*(*s*) and log *s*, and the slope of this relationship corresponds to the FD [17].

$${{\mbox{Log}}}{{N}}({\mbox{s}})\,=\,-{{D}}_{{\mbox{f}}}\,{\mbox{Logs}}\,+\,{c}$$

*D* represents the fractal dimension, and *C* is a constant. For a two-dimensional endocardial contour, its value is a non-integer between 1 and 2; the closer it is to 2, the more pronounced the edge tortuosity, which can indirectly reflect pathological remodeling of myocardial tissue morphology [18].

### Biventricular perimeter-area ratio (PAR)

PAR provides a scale-dependent measure of shape compactness. For each segmented myocardial region, we calculated:

$${{\mbox{PAR}}}\,=\,\frac{{P}}{{A}}$$

*P* denotes the contour perimeter, and *A* represents the enclosed area. Connected component labeling was first applied to identify discrete regions, and measurements were obtained from the primary (largest) connected component [16].

### Right heart catheterization (RHC)

All CPTE patients underwent RHC via either the right internal jugular or femoral vein using a 6 F Swan-Ganz catheter (Bioptimal). The following hemodynamic parameters were measured: Mean pulmonary arterial pressure (mPAP), systolic pulmonary arterial pressure (sPAP), diastolic pulmonary arterial pressure (dPAP), mean right atrial pressure (mRAP), mean right ventricular pressure (mRVP), systolic right ventricular pressure (sRVP), diastolic right ventricular pressure (dRVP), pulmonary artery wedge pressure (PAWP), pulmonary vascular resistance (PVR), pulmonary vascular resistance index (PVRI) and mixed venous oxygen saturation (SvO2). Cardiac output (CO) and cardiac index (CI) were calculated using the Fick method. According to RHC, patients were classified as the CTEPH group if mPAP ≥ 20 mmHg, PAWP ≤ 15mmHg, and PVR > 2 WU; otherwise, they were classified as the CTEPD group.

### Statistical analysis

Statistical analyses were performed using SPSS 27.0 (Chicago, IL, USA) and R 4.3.3. Measurement reproducibility was assessed using a Bland–Altman plot and intraclass correlation coefficients (ICC). Diagnostic performance was evaluated by receiver operating characteristic (ROC) curve analysis, with optimal cut-off values determined by the Youden index. Bootstrap internal validation (1000 resamples) was performed for all ROC analyses. Multivariable logistic regression was used to construct predictive models. The net reclassification improvement (NRI) and integrated discrimination improvement (IDI) quantified the incremental clinical value of morphological parameters beyond conventional indices. Normal data were expressed as mean ± standard deviation (SD), and one-way ANOVA and independent-sample *T*-test were used for comparison in different groups; non-normal data were expressed as median with inter-quartile range (IQR), and Kruskal-Wallis test and Mann–Whitney *U*-test were used for comparison among three groups. Count data were expressed as frequency (percentage), and *χ*^2^ test was used for comparison in different groups. Spearman’s rank correlation analysis was used to assess the correlations of morphological parameters with hemodynamic indices and disease duration, with correlation strengths categorized as strong (|*ρ* | ≥ 0.7), moderate (0.5–0.7), weak (0.3–0.5), and negligible (< 0.3). Multivariable linear regression was used to control for confounding factors. A *p*-value of less than 0.05 was considered statistically significant.

## Results

### Clinical characteristics

53 CPTE patients (29 males, mean age = 53.30 ± 15.99 years old) were included, comprising 16 CTEPD patients (7 males, mean age =  45.56 ± 17.05 years old) and 37 CTEPH patients (22 males, mean age = 56.65 ± 14.49 years old), along with 53 healthy controls (29 males, mean age = 52.89 ± 7.86 years old). Baseline clinical characteristics are summarized in Tables 1 and 2. No significant differences were observed between the CPTE and controls in sex, age, height, or body surface area (BSA) (*p* > 0.05). Compared with both the CTEPD and controls, the CTEPH group had significantly higher N-terminal pro-B-type natriuretic peptide (NT-proBNP) and D-dimer levels and lower systolic blood pressure (SBP) (*p* < 0.05). Diastolic blood pressure (DBP) was lower in the CTEPH and CTEPD groups than in the control group (*p* < 0.05).

**Table 1:** Baseline characteristics of CPTE and healthy controls

Characteristic	Group A (*n* = 53)	Group B (*n* = 53)	*t*/*χ*²/*Z* (*p*)
Age (years old)	53.30 ± 15.99	52.89 ± 7.86	0.17 (0.866)
Gender (male/female)	29/24	29/24	0.00 (1.000)
Height (m)	1.67 ± 0.07	1.67 ± 0.07	0.17 (0.864)
Weight (kg)	69.13 ± 12.53	74.81 ± 10.66	2.51 (0.014)*
BMI (kg/m^2^)	24.54 ± 3.50	26.64 ± 2.64	3.49 (< 0.001)*
BSA (m^2^)	1.77 ± 0.18	1.84 ± 0.15	1.91 (0.058)
SBP (mmHg)	117.00 ± 15.49	130.81 ± 13.83	4.84 (< 0.001)*
DBP (mmHg)	76.06 ± 12.64	88.60 ± 10.71	5.51 (< 0.001)*
NT-proBNP (pg/mL)	181 (34.5, 907)	29.5 (10, 37.75)	-5.27 (< 0.001)*
D-dimer (mg/L)	0.25 (0.14, 0.75)	0.16 (0.15, 0.23)	-2.48 (0.013)*

Group A: *CPTE* Chronic pulmonary thromboembolism; Group B: healthy controls; * *p* < 0.05; *BMI* Body mass index, *BSA* Body surface area, *SBP* Systolic blood pressure, *DBP* Diastolic blood pressure, *NT pro-BNP* N-Terminal pro-brain natriuretic peptide

**Table 2:** Baseline characteristics and hemodynamic characteristics among the three groups

Characteristics	Group A (*n* = 37)	Group B (*n *= 16)	Group C (*n* = 53)	*F*/*t* /*χ*²(*P*)	A and B	A and C	B and C
Age (years old)	56.65 ± 14.49	45.56 ± 17.05	52.89 ± 7.86	4.68 (0.011)*	0.093	0.400	0.305
Gender (male/female)	22/15	7/9	29/24	1.11 (0.573)	0.372	0.672	0.570
Height (m)	1.67 ± 0.08	1.68 ± 0.06	1.67 ± 0.07	0.07 (0.927)	0.728	0.994	0.711
Weight (kg)	66.48 ± 11.15	75.25 ± 13.73	74.81 ± 10.66	6.66 (0.002)*	0.011*	< 0.001*	0.892
BMI (kg/m^2^)	23.70 ± 3.19	26.48 ± 3.51	26.64 ± 2.64	11.46 (< 0.001)*	0.002*	< 0.001*	0.848
BSA (m^2^)	1.74 ± 0.17	1.85 ± 0.18	1.84 ± 0.15	4.15 (0.018)*	0.036*	0.009*	0.818
SBP (mmHg)	113.91 ± 15.72	124.12 ± 12.70	130.81 ± 13.83	15.05 (< 0.001)*	0.019*	< 0.001*	0.106
DBP (mmHg)	74.21 ± 13.26	80.31 ± 10.20	88.60 ± 10.71	17.04 (< 0.001)*	0.082	< 0.001*	0.014*
NT-proBNP (pg/mL)	446 (57, 1388)	36.5 (14.5, 97.5)	29.5 (10, 37.75)	40.30 (< 0.001)*	0.001*	0.000*	0.646
D-dimer (mg/L)	0.45 (0.21, 1.06)	0.13 (0.10, 0.16)	0.16 (0.15, 0.23)	32.37 (< 0.001)*	0.000*	0.000*	0.189
Disease duration (months)	19 (5.5, 29)	12.5 (5.25, 23)	-	0.85	0.393	-	-
sPAP (mmHg)	69.89 ± 20.81	26.75 ± 5.10	-	11.75	< 0.001*	-	-
dPAP (mmHg)	23.11 ± 8.53	10.44 ± 2.70	-	8.24	< 0.001*	-	-
mPAP (mmHg)	40.00 ± 12.06	16.88 ± 3.34	-	10.71	< 0.001*	-	-
PAWP (mmHg)	10.03 ± 3.26	9.69 ± 2.44	-	0.37	0.711	-	-
CO (L/min)	3.40 (2.84, 3.96)	5.03 (4.41, 6.08)	-	-3.99	< 0.001*	-	-
CI (L/min/m^2^)	2.00 (1.66, 2.26)	2.83 (2.29, 3.23)	-	-3.93	< 0.001*	-	-
PVR (wood)	8.14 (5.76, 13.02)	1.40 (1.05, 2.00)	-	5.02	< 0.001*	-	-
PVRI (wood units·m^2^)	14.10 (10.27, 21.60)	2.50 (1.94, 3.77)		4.96	< 0.001*		
mRAP (mmHg)	4.00 (2.00, 6.00)	3.00 (2.25, 5.75)	-	0.17	0.860	-	-
sRVP (mmHg)	69.92 ± 23.38	27.56 ± 5.91	-	10.28	< 0.001*	-	-
dRVP (mmHg)	2.00 (1.00, 4.00)	2.00 (0.25, 4.00)	-	0.59	0.551	-	-
mRVP (mmHg)	25.14 ± 9.46	11.88 ± 2.41	-	8.03	< 0.001*	-	-
SvO2 (%)	67.79 ± 7.08	77.30 ± 3.95	-	-5.09	< 0.001*	-	-

Group A: *CTEPH* Chronic thromboembolic pulmonary hypertension; Group B: *CTEPD* Chronic thromboembolic pulmonary disease; Group C: healthy controls; * *p* < 0.05

*BMI* Body mass index, *BSA* Body surface area, *SBP* Systolic blood pressure, *DBP* Diastolic blood pressure, *NT pro-BNP* N-Terminal pro-brain natriuretic peptide, *mPAP* Mean pulmonary arterial pressure, *sPAP* Systolic pulmonary arterial pressure, *dPAP* Diastolic pulmonary arterial pressure, *mRAP* Mean right atrial pressure, *mRVP* Mean right ventricular pressure, *sRVP* Systolic right ventricular pressure, *dRVP* Diastolic right ventricular pressure, *PAWP* Pulmonary artery wedge pressure, *PVR* Pulmonary vascular resistance, *PVRI* Pulmonary vascular resistance index, *SvO2* Mixed venous oxygen saturation, *CO* Cardiac output, *CI* Cardiac index

As shown in Table 2, the CTEPH group had significantly higher values of sPAP, dPAP, mPAP, PVR, PVRI, sRVP, and mRVP, along with significantly lower CO, CI, and SvO₂ compared to the CTEPD group (all *p* < 0.001). No significant differences were found between the two groups regarding PAWP, mRAP, dRVP and disease duration.

### Biventricular functional parameters among the three groups

As shown in Table 3, right ventricular ejection fraction (RVEF) progressively decreased from the controls to the CTEPD and further to the CTEPH, with statistically significant differences among all three groups (*p* < 0.05). Right ventricular end-diastolic volume index (RVEDVI) and right ventricular end-systolic volume index (RVESVI) were significantly higher in the CTEPH group than in both the CTEPD and controls (*p* < 0.05).

**Table 3:** Cardiac function parameters among the three groups

Function	Group A (*n* = 37)	Group B (*n* = 16)	Group C (*n* = 53)	*H* (*p*)	A and B	A and C	B and C
RVEF (%)	40.71 (33.78, 46.67)	47.66 (44.77, 56.41)	57.24 (53.02, 61.65)	54.26 (< 0.001)*	0.039*	0.000*	0.011*
RVEDVI (mL/m^2^)	90.85 (73.17, 106.83)	55.38 (46.70, 69.77)	42.74 (34.37, 52.55)	52.47 (< 0.001)*	0.005*	0.000*	0.105
RVESVI (mL/m^2^)	51.14 (41.58, 63.40)	26.31 (23.35, 35.51)	17.11 (14.15, 23.52)	57.76 (< 0.001)*	0.014*	0.000*	0.019*
LVEF (%)	64 (56, 67)	60.5 (56.25, 65.5)	68 (63, 71)	13.76 (0.001)*	1.000	0.013*	0.005*
LVEDVI (mL/m^2^)	61.22 (48.86, 72.70)	69.74 (51.59, 78.36)	57.00 (52.50, 62.90)	3.10 (0.211)	0.405	0.299	0.093
LVESVI (mL/m^2^)	22.99 (14.93, 34.28)	24.98 (17.70, 34.93)	19.00 (16.00, 22.50)	9.42 (0.009)*	1.000	0.050*	0.031*

Group A: *CTEPH* Chronic thromboembolic pulmonary hypertension; Group B: *CTEPD* Chronic thromboembolic pulmonary disease; Group C: Healthy controls; * *p* < 0.05; *LVEF* Left ventricular ejection fraction, *LVEDVI* Left ventricular end-diastolic volume index, *LVESVI* Left ventricular end-systolic volume index, *RVEF* Right ventricular ejection fraction, *RVEDVI* Right ventricular end-diastolic volume index, *RVESVI* Right ventricular end-systolic volume index

Left ventricular ejection fraction (LVEF) was significantly higher in controls than in the CTEPH and CTEPD groups (*p* < 0.05), while left ventricular end-systolic volume index (LVESVI) was significantly lower in controls than in the CTEPH and CTEPD groups (*p* < 0.05). Left ventricular end-diastolic volume index (LVEDVI) was similar among the three groups.

### Comparison of biventricular morphological parameters among the three groups

As shown in Table 4, RVFD in both ES and ED increased progressively from controls to CTEPD and to CTEPH. The most significant intergroup differences were observed at the apical level (*p *< 0.05), with the lowest values in controls, intermediate in CTEPD, and the highest in CTEPH. At the basal and papillary muscle levels in ED, no significant difference was observed between CTEPD and CTEPH. At the papillary muscle level in ES, CTEPH differed significantly from controls; however, no significant difference was found at the basal level.

**Table 4:** Comparison of biventricular FD and PAR among the three groups

Variables	Group A (*n* = 37)	Group B (*n* = 16)	Group C (*n* = 53)	*H* (*p*)	A and B	A and C	B and C
RVFD							
d1	1.252 (1.242, 1.258)	1.250 (1.244, 1.260)	1.244 (1.234, 1.249)	12.70 (0.002)*	1.000	0.008*	0.017*
d2	1.256 (1.250, 1.266)	1.250 (1.247, 1.258)	1.235 (1.224, 1.243)	50.62 (< 0.001)*	1.000	0.000*	0.000*
d3	1.258 (1.250, 1.266)	1.240 (1.228, 1.250)	1.219 (1.208, 1.229)	66.56 (< 0.001)*	0.030*	0.000*	0.002*
s1	1.235 (1.222, 1.242)	1.227 (1.216, 1.237)	1.225 (1.216, 1.236)	3.38 (0.185)	0.233	0.077	0.949
s2	1.240 (1.233, 1.250)	1.233 (1.223, 1.241)	1.228 (1.215, 1.240)	11.98 (0.002)*	0.220	0.002*	1.000
s3	1.249 (1.242, 1.255)	1.230 (1.219, 1.244)	1.211 (1.190, 1.222)	61.53 (< 0.001)*	0.012*	0.000*	0.013*
RVPAR							
d1	0.138 (0.127, 0.158)	0.149 (0.141, 0.154)	0.185 (0.179, 0.208)	68.93 (< 0.001)*	1.000	0.000*	0.000*
d2	0.145 (0.126, 0.163)	0.163 (0.153, 0.170)	0.229 (0.212, 0.270)	74.46 (< 0.001)*	0.246	0.000*	0.000*
d3	0.150 (0.142, 0.180)	0.196 (0.179, 0.229)	0.298 (0.252, 0.355)	74.66 (< 0.001)*	0.016*	0.000*	0.001*
s1	0.172 (0.151, 0.189)	0.211 (0.183, 0.228)	0.234 (0.211, 0.258)	51.15 (< 0.001)*	0.013*	0.000*	0.052
s2	0.157 (0.140, 0.191)	0.214 (0.194, 0.234)	0.267 (0.229, 0.296)	66.45 (< 0.001)*	0.003*	0.000*	0.022*
s3	0.172 (0.145, 0.193)	0.223 (0.213, 0.305)	0.355 (0.283, 0.446)	71.38 (< 0.001)*	0.011*	0.000*	0.003*
LVFD							
d1	1.211 (1.197, 1.214)	1.208 (1.202, 1.212)	1.204 (1.193, 1.210)	5.80 (0.055)	0.899	0.043	0.056
d2	1.211 (1.197, 1.219)	1.214 (1.204, 1.219)	1.204 (1.193, 1.213)	6.86 (0.032)*	1.000	0.169	0.065
d3	1.209 (1.196, 1.215)	1.208 (1.196, 1.217)	1.190 (1.180, 1.204)	22.53 (< 0.001)*	1.000	0.000*	0.004*
s1	1.187 (1.179, 1.199)	1.188 (1.179, 1.198)	1.178 (1.169, 1.191)	4.64 (0.098)	0.977	0.056	0.124
s2	1.179 (1.170, 1.190)	1.183 (1.179, 1.189)	1.178 (1.169, 1.190)	1.71 (0.423)	0.195	0.905	0.248
s3	1.175 (1.158, 1.193)	1.179 (1.159, 1.185)	1.174 (1.154, 1.186)	0.80 (0.669)	0.690	0.378	0.754
LVPAR							
d1	0.194 (0.165, 0.254)	0.203 (0.171,0.281)	0.256 (0.219, 0.306)	17.89 (< 0.001)*	1.000	0.000*	0.034*
d2	0.255 (0.215, 0.293)	0.252 (0.233, 0.302)	0.288 (0.261, 0.342)	12.44 (0.002)*	1.000	0.006*	0.031*
d3	0.251 (0.239, 0.275)	0.261 (0.237, 0.318)	0.293 (0.260, 0.332)	16.97 (< 0.001)*	1.000	0.000*	0.029*
s1	0.287 (0.238, 0.339)	0.245 (0.224, 0.283)	0.332 (0.287, 0.369)	15.59 (< 0.001)*	0.361	0.025*	0.001*
s2	0.412 (0.302, 0.491)	0.325 (0.291, 0.372)	0.377 (0.310, 0.460)	2.48 (0.289)	0.157	0.724	0.147
s3	0.438 (0.389, 0.539)	0.398 (0.354, 0.487)	0.407 (0.316, 0.574)	1.25 (0.535)	0.269	0.410	0.744

Group A: *CTEPH* Chronic thromboembolic pulmonary hypertension, Group B: *CTEPD* Chronic thromboembolic pulmonary disease, Group C: healthy controls; * *p* < 0.05; *d1* End-diastolic basal level, *d2* End-diastolic papillary muscle level, *d3* End-diastolic apical level, *s1* End-systolic basal level, *s2* End-systolic papillary muscle level, *s3* End-systolic apical level, *RVFD* Right ventricular fractal dimension, *RVPAR* Right ventricular perimeter-area ratio, *LVFD* Left ventricular fractal dimension, *LVPAR* Left ventricular perimeter-area ratio

RVPAR progressively decreased with disease severity, which was highest in controls, intermediate in CTEPD, and lowest in CTEPH, again with the most marked differences at the apical level in ED and ES (*p* < 0.05). Moreover, RVPAR at the papillary muscle level in ES was also highest in controls, intermediate in CTEPD, and lowest in CTEPH.

RVPAR in ED between CTEPH and CTEPD did not differ significantly at the basal and papillary muscle levels, nor between CTEPD and controls at the basal level in ES (*p* > 0.05).

For the left ventricle, at the apical level, LVFD in ED was significantly higher in both CTEPH and CTEPD groups compared to controls (*p* < 0.05), but did not differ between CTEPH and CTEPD. LVFD at other levels showed no significant intergroup differences. At all levels in ED and at the basal level in ES, LVPAR was significantly lower in CTEPH and CTEPD groups than in controls (*p* < 0.05), with no significant difference between CTEPD and CTEPH groups.

### Reproducibility assessment

As shown in Supplementary Table S1, a stratified random sample of 32 cases (≈30%) was selected for validation. Baseline characteristics did not differ significantly between this subsample and the overall cohort (*p* > 0.05). Intra-observer reproducibility was assessed by the same radiologist with 5 years of cardiovascular imaging experience (interval ≥ 2 weeks). Inter-observer reproducibility was evaluated by a second cardiovascular radiologist with 5 years of experience who was blinded to clinical data. As shown in Supplementary Table S2, intra-observer ICCs ranged from 0.738 to 0.964. As shown in Supplementary Table S3, inter-observer ICCs ranged from 0.732 to 0.945. Both FD and PAR demonstrated good to excellent reproducibility across all ventricular levels.

**Fig. 3:**
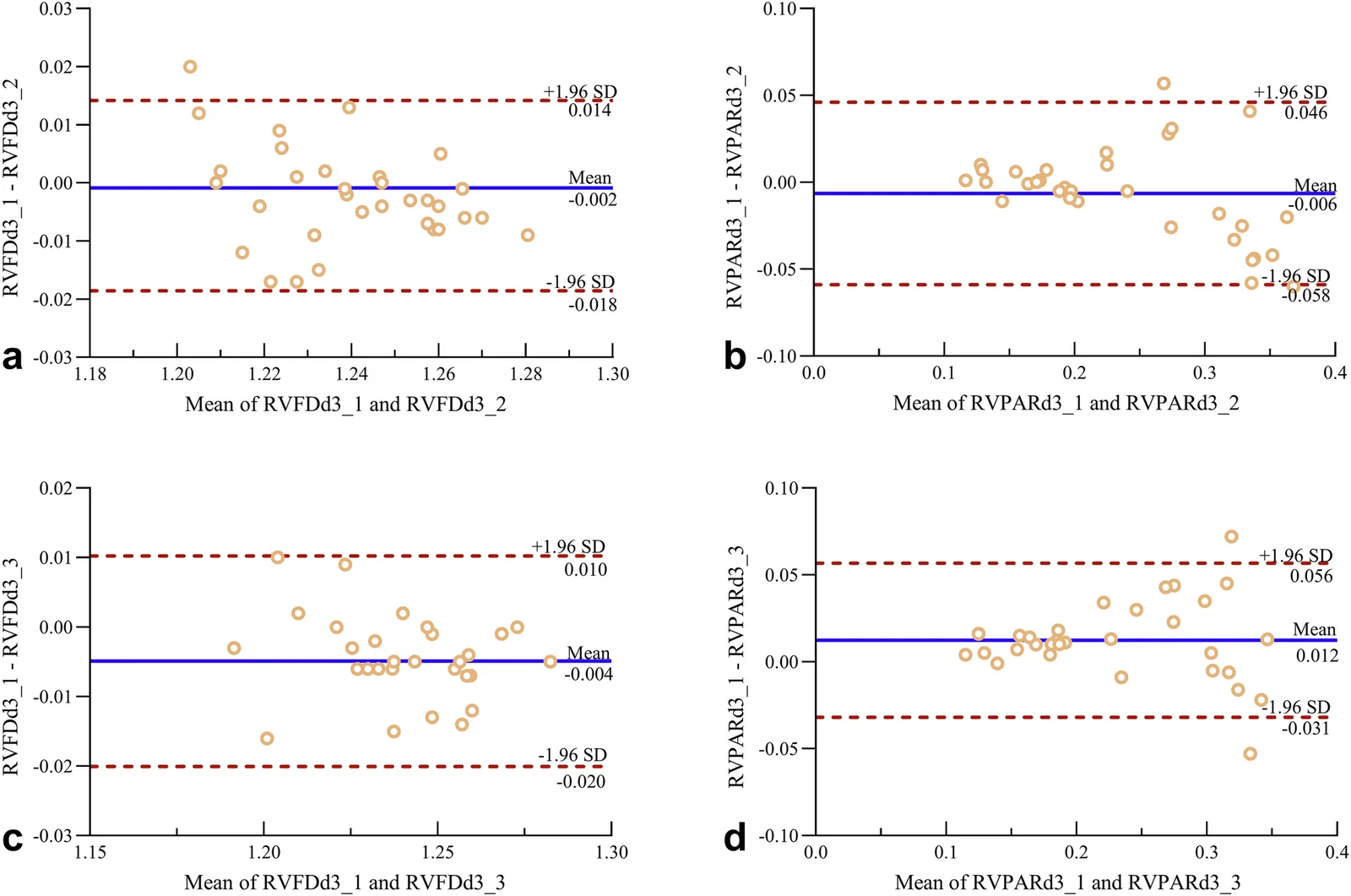
Bland-Altman plots at the right ventricular end-diastolic apical level.** a** The intra-observer for RVFD; **b** The intra-observer for RVPAR; **c** The inter-observer for RVFD; **d** The inter-observer for RVPAR. RVFD, Right ventricular fractal dimension; RVPAR, Right ventricular perimeter-area ratio

Bland-Altman analysis at the right ventricular apical level in end-diastole (Fig. 3) showed clinically acceptable limits of agreement. For the same observer, the mean bias for RVFD was -0.002 (+ 1.96 SD: 0.014; -1.96 SD: -0.018); for RVPAR, the mean bias was -0.006 (+ 1.96 SD: 0.046; -1.96 SD: -0.058). For the two observers, the mean bias for RVFD was -0.004 (+ 1.96 SD: 0.010; -1.96 SD: -0.020); for RVPAR, the mean bias was 0.012 (+ 1.96 SD: 0.056; -1.96 SD: -0.031).

### Diagnostic performance and incremental diagnostic value

As shown in Table 5, at the apical level, the best discrimination was observed between CTEPH and controls. In ED, RVFD (cutoff 1.238) yielded a sensitivity of 97.3% and specificity of 88.6%, with an area under the curve (AUC) (bootstrap 95% CI) of 0.975 (0.918–0.993); RVPAR (cutoff 0.209) achieved a sensitivity of 97.3% and specificity of 100%, with an AUC of 0.990 (0.936–1.000). In ES, RVFD (cutoff 1.223) yielded a sensitivity of 100% and specificity of 75.4%, with an AUC (bootstrap 95% CI) of 0.959 (0.914–0.985); RVPAR (cutoff 0.257) achieved a sensitivity of 94.5% and specificity of 94.3%, with an AUC of 0.979 (0.932–0.998). For distinguishing CPTE from controls, AUCs ranged from 0.911 to 0.961, with sensitivities of 84.9–90.5% and specificities of 75.4–100%. In differentiating CTEPD from controls, AUCs ranged from 0.798 to 0.892, with RVFD reaching 100% sensitivity. For distinguishing CTEPH from CTEPD, AUCs ranged from 0.819 to 0.895, with RVPAR achieving 100% sensitivity.

**Table 5:** Receiver Operating Characteristic Curves among the three groups

Groups	Cut-off value	AUC (Bootstrap 95%CI)	*p*-value	Sensitivity%	Specificity%	Youden Index
CPTE-Healthy						
RVFDd3	> 1.236	0.936 (0.871, 0.966)	< 0.0001	84.9	88.6	0.735
RVPARd3	< 0.214	0.961 (0.899, 0.989)	< 0.0001	88.6	100	0.886
RVFDs3	> 1.222	0.911 (0.844, 0.949)	< 0.0001	90.5	75.4	0.660
RVPARs3	< 0.257	0.947 (0.900, 0.976)	< 0.0001	84.9	94.3	0.792
CTEPH-Healthy						
RVFDd3	> 1.238	0.975 (0.918, 0.993)	< 0.0001	97.3	88.6	0.859
RVPARd3	< 0.209	0.990 (0.936, 1.000)	< 0.0001	97.3	100	0.973
RVFDs3	> 1.223	0.959 (0.914, 0.985)	< 0.0001	100	75.4	0.754
RVPARs3	< 0.257	0.979 (0.932, 0.998)	< 0.0001	94.5	94.3	0.889
CTEPD-Healthy						
RVFDd3	> 1.222	0.846 (0.728, 0.925)	< 0.0001	100	56.6	0.566
RVPARd3	< 0.231	0.892 (0.707, 0.971)	< 0.0001	81.2	92.4	0.737
RVFDs3	> 1.220	0.798 (0.668, 0.893)	0.0003	75	71.7	0.467
RVPARs3	< 0.243	0.872 (0.768, 0.950)	< 0.0001	62.5	96.2	0.587
CTEPD-CTEPH						
RVFDd3	< 1.252	0.819 (0.616, 0.918)	0.0003	81.2	72.9	0.542
RVPARd3	> 0.164	0.887 (0.752, 0.951)	< 0.0001	100	64.8	0.648
RVFDs3	< 1.246	0.838 (0.681, 0.930)	< 0.0001	64.8	87.5	0.523
RVPARs3	> 0.204	0.895 (0.766, 0.959)	< 0.0001	83.7	93.7	0.775

*CPTE* Chronic pulmonary thromboembolism, *CTEPH* Chronic thromboembolic pulmonary hypertension, *CTEPD* Chronic thromboembolic pulmonary disease, *RVFD* Right ventricular fractal dimension, *RVPAR* Right ventricular perimeter-area ratio, *LVFD* Left ventricular fractal dimension, *LVPAR* Left ventricular perimeter-area ratio, *d3* End-diastolic apical level, *s3* End-systolic apical level

To evaluate the incremental diagnostic value of geometric morphological parameters over conventional cardiac function indices, a multivariable logistic regression model was built to discriminate healthy controls from CPTE patients. The baseline model included only RVEF to avoid collinearity, while the extended models respectively incorporated apical RVFD and RVPAR in ED. Variance inflation factor (VIF) confirmed that the geometric parameters were independent of RVEF. Bootstrap-derived AUCs (95% CI) were as follows: baseline model 0.891 (0.810–0.946); adding RVFD 0.967 (0.900–0.988); adding RVPAR 0.973 (0.901–0.993). DeLong’s test showed that both extended models had significantly higher AUCs than the baseline model (*p* < 0.01). NRI and IDI further supported the incremental value: for RVFD, continuous NRI = 1.396, categorical NRI = 0.434, IDI = 0.247 (all *p* < 0.001); for RVPAR, continuous NRI = 1.321, categorical NRI = 0.547, IDI = 0.263 (all *p* < 0.001).

To assess whether RVPAR provides diagnostic information beyond RVEDVI, a logistic regression model containing both was constructed. The VIF was 1.400. The AUC was 0.867 (0.799–0.935) for RVEDVI alone and 0.960 (0.921–0.996) after adding RVPAR (*p* = 0.0043).

### Correlation of geometric morphological parameters and hemodynamics in CPTE patients

**Fig. 4:**
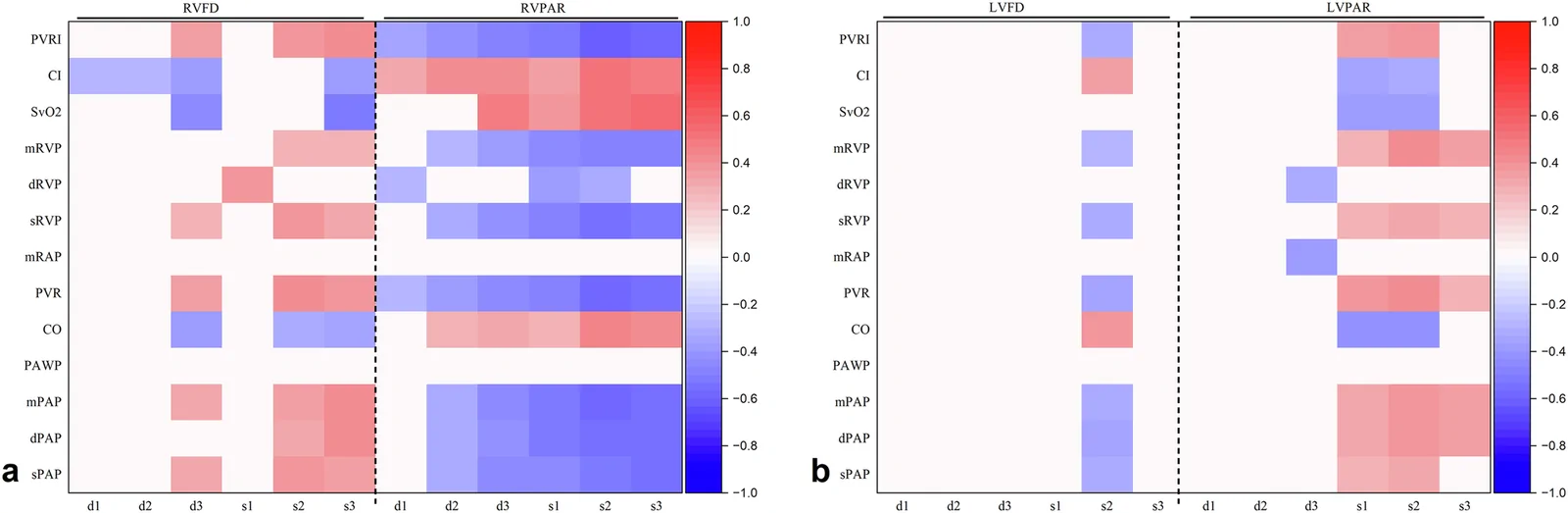
**a** Correlation analysis of right ventricular morphological parameters; **b** Correlation analysis of left ventricular morphological parameters. mPAP, Mean pulmonary arterial pressure; sPAP, Systolic pulmonary arterial pressure; dPAP, Diastolic pulmonary arterial pressure; mRAP, Mean right atrial pressure; mRVP, Mean right ventricular pressure; sRVP, Systolic right ventricular pressure; dRVP, Diastolic right ventricular pressure; PAWP, Pulmonary artery wedge pressure; PVR, Pulmonary vascular resistance; PVRI, Pulmonary vascular resistance index; SvO2, Mixed venous oxygen saturation; CO, Cardiac output; CI, Cardiac index; d1, End-diastolic basal level; d2, End-diastolic papillary muscle level; d3, End-diastolic apical level; s1:, End-systolic basal level; s2, End-systolic papillary muscle level; s3, End-systolic apical level

In CPTE patients (Fig. 4), apical RVFD in ED showed weak positive correlation with PVR, PVRI, sPAP, mPAP, sRVP (*ρ* = 0.278 to 0.362, all *p* < 0.05), and weak negative correlations with SvO₂, CO, and CI (*ρ* = -0.375 to -0.445, all *p* < 0.01). At papillary muscle and apical levels, RVFD in ES showed weak positive correlations with PVR, PVRI, sPAP, mPAP, dPAP, sRVP, and mRVP (*ρ* = 0.283 to 0.469, all *p* < 0.05), and moderate-to-weak negative correlations with SvO₂, CO, and CI (*ρ* = -0.329 to -0.522, all *p* < 0.05).

At the papillary muscle and apical levels, RVPAR in ED showed weak negative correlations with PVR, PVRI, mPAP, sPAP, dPAP, sRVP, and mRVP (*ρ* = -0.308 to -0.491, all *p* < 0.05), and weak positive correlations with SvO₂, CO, and CI (*ρ* = 0.298 to 0.488, all *p* < 0.05). These correlations strengthened in ES across all levels: moderate-to-weak negative correlations with PVR, PVRI, mPAP, sPAP, dPAP, sRVP, mRVP, and dRVP (*ρ* = -0.316 to -0.604, all *p* < 0.05), and moderate-to-weak positive correlations with SvO₂, CO, and CI (*ρ* = 0.272 to 0.552, all *p* < 0.05).

At the papillary muscle level, LVFD in ES showed weak positive correlations with CO (*ρ* = 0.375, *p* < 0.01) and CI (*ρ* = 0.334, *p* < 0.05), and weak negative correlations with PVR, PVRI, sPAP, dPAP, mPAP, sRVP, and mRVP (*ρ* = -0.284 to -0.360, all *p* < 0.05). LVPAR in ES showed weak positive correlations with PVR, PVRI, sPAP, dPAP, mPAP, sRVP, and mRVP (*ρ* = 0.278 to 0.425, all *p* < 0.05), and weak negative correlations with SvO₂, CO, and CI (*ρ* = -0.339 to -0.420, all *p* < 0.05).

### Correlation with disease duration and age

The disease duration from diagnosis to CMR was analyzed. At the apical levels, only RVFD in ES showed a weak negative correlation with disease duration (*ρ* = -0.286, *p* = 0.038); no other apical geometric parameter was significantly correlated (all *p* > 0.05). This correlation did not remain significant after adjusting for age and RVEF in multivariable linear regression (*p* > 0.05).

Spearman correlation analysis was also performed to assess the relationship between age and apical geometric parameters. Age showed a moderate positive correlation with RVFD in ED (*ρ* = 0.432, *p* = 0.001), but no significant correlations with RVFD in ES, or with RVPAR in ED or ES (all *p* > 0.05).

### Multivariable linear regression analyses

To control for confounding factors, multivariable linear regression models were constructed to assess the independent associations of age, apical geometric parameters and RVEF with PVR and mPAP. As shown in Supplementary Tables S4 and S5, age and RVEF were independent predictors of hemodynamic indices in all models (*p* < 0.05). RVFD was not independently associated with either PVR or mPAP. RVPAR showed an independent negative association with mPAP in ED (*β* = -0.208, *p* = 0.040) and with PVR in ES (*β* = -0.251, *p* = 0.041).

## Discussion

In this pilot study, we analyzed biventricular FD and PAR characteristics in CPTE patients, and there were several interesting findings: (I) CMR-based RVFD increases in CTEPD and CTEPH patients, with the most significant differences observed at the RV apical region; (II) CMR-based RVPAR decreases in CTEPD and CTEPH patients, also with the most significant differences observed at the apical region. (III) CMR-based RVFD and RVPAR at the apical region demonstrate excellent subtype differentiation; (IV) Both FD and PAR provide significant incremental diagnostic value beyond conventional RVEF, with RVPAR being slightly superior.

Myocardial trabeculae form the anatomical basis for endocardial contour complexity [18]. Their development, beginning in embryogenesis, is regulated by hemodynamic shear stress and myocardial strain, exhibiting racial variation and load adaptability [19]. Prior studies indicated trabeculae were sensitive to microstructural remodeling and were closely linked to pulmonary vascular afterload, RV function, and prognosis [12]. In CPTE, chronic pulmonary pressure overload induces RV hypertrophy accompanied by trabecular thickening and fibrosis, thereby increasing endocardial surface complexity and elevating FD [20]. This observation aligns with Dawes et al’s findings on increased RV trabecular complexity in PH [12]. With the progressive increase in pressure load, there is further RV eccentric dilation and an increase in RV area. Since the rate of increase in RV area exceeds that of RV perimeter, RVPAR decreases. This change reflects the morphological transformation of the ventricle from “compact” to “dilated”. The distinct RVFD and RVPAR trends reflect the dual processes of cardiac remodeling: myocardial hypertrophy (trabeculation) and chamber dilation, collectively enabling a more comprehensive, multidimensional characterization.

Notably, among the control group, RVFD and RVPAR are most pronounced at the apical level, suggesting that right ventricular remodeling occurs in CTEPD even without pulmonary hypertension, and that RV remodeling may occur earliest in the apical region. Moreover, apical RVFD and RVPAR provided significant incremental diagnostic value over conventional CMR functional indices, indicating that FD and PAR may capture early myocardial structural remodeling that conventional parameters may miss. The apical sensitivity likely arises from the region having the densest myocardial trabeculae, making it highly responsive to hemodynamic changes [21]. Under pressure overload, the trabeculae at the apex of the heart are more likely to undergo structural remodeling. The myocardium in the apex experiences the highest blood flow-induced wall shear stress, thus exhibiting the most significant changes during systole and diastole. Moreover, the cardiac apex is the “power endpoint” of ventricular pumping, and changes in its morphology more directly reflect ventricular remodeling.

Furthermore, we analyzed the correlation of RVFD and RVPAR with hemodynamics. In univariable analysis, RVFD correlated positively with afterload indices and negatively with perfusion/output metrics, while RVPAR showed opposite correlations. These findings suggest that greater RV endocardial complexity is associated with higher pulmonary vascular resistance and reduced cardiac output [22]. Notably, RVPAR showed stronger and more consistent hemodynamic associations than FD, particularly during systole. However, after adjusting for age and RVEF in multivariable linear regression, RVFD was no longer independently associated with PVR or mPAP, whereas RVPAR showed independent negative associations with mPAP and PVR. These findings suggest that the univariable associations of RVFD are largely driven by age and RV dysfunction, whereas RVPAR may capture additional aspects of ventricular remodeling beyond conventional parameters.

Although the LV is relatively indirectly affected by the pulmonary circulation, subtle increases in LVFD and reductions in LVPAR were observed, particularly in apical slices. These changes showed weaker but plausible correlations with hemodynamic measures, suggesting septal-mediated ventricular interdependence [23]. Future studies could explore the potential value of these morphological parameters in evaluating left heart diseases.

This study has several limitations. First, this single-center study at a tertiary referral hospital may introduce selection bias, and the modest sample size, particularly the CTEPD subgroup (*n* = 16), limits statistical power and generalizability to community populations or earlier disease stages. Multicenter validation in larger, broader cohorts is therefore needed. Second, although bootstrap validation was applied to the AUCs, the optimal cut-off values and their corresponding sensitivities and specificities were derived from the same cohort using the Youden index and are therefore optimistic. In particular, the 100% sensitivities observed in some comparisons should be interpreted with caution. External confirmation in independent cohorts is essential before clinical application. Additionally, although the age difference between CTEPH and CTEPD did not reach statistical significance (*p* = 0.093), the numerical difference is clinically relevant. Age correlated with RVFD in ED, which may modestly affect the diagnostic performance of RVFD for subtype differentiation; therefore, future age-adjusted analyses are warranted. Third, all patients in this study received stable anticoagulation therapy. Due to the small sample size in the warfarin group (*n* = 5), no formal statistical comparison of imaging findings across different treatment regimens was performed. Future multicenter studies with larger sample sizes are needed to evaluate the effects of different anticoagulant agents on right ventricular remodeling. Finally, multivariable linear regression showed that apical RVFD had no independent linear association with continuous hemodynamic indices, and its univariable correlations were largely mediated by age and RV function. Although apical RVPAR demonstrated weak independent negative correlations with mPAP and PVR, the effect sizes were small. The correlation between disease duration and geometric parameters also became non-significant after multivariable adjustment. Nevertheless, in diagnostic classification tasks, geometric parameters exhibited significant incremental value, suggesting that they may improve diagnostic accuracy by capturing non-linear patterns or complementary information dimensions relative to conventional functional indices, rather than through independent linear dose-response relationships. Regarding RVPAR, although it correlated with RVEDVI, a logistic model containing both showed no prohibitive collinearity, and RVPAR still provided additional diagnostic value beyond RVEDVI alone. This suggests that its incremental value may not be fully explained by dilation alone, and may partly reflect morphological complexity beyond volume. However, given the exploratory nature of this analysis and the observed correlation with RVEDVI, future studies using volume-adjusted indices or larger cohorts are needed to further validate its independent contribution.

Collectively, these findings indicate that the clinical interpretation of geometric parameters should be integrated with conventional functional indices, and their independent prognostic value and underlying mechanisms warrant further validation in larger longitudinal studies. Accordingly, we are currently planning a prospective follow-up of CTEPD patients to observe right ventricular remodeling over time and validate the hypothesized apical-first remodeling pattern.

## Conclusion

Geometric morphological parameters derived from CMR demonstrated potential to detect subtle myocardial structural remodeling. The apical region, owing to its rich trabecular architecture and unique hemodynamic environment, is the most sensitive area for morphological remodeling. The pronounced differences and incremental value of RVFD and RVPAR at the apical level indicate that they could complement existing CMR assessment, offering a novel imaging perspective for early CPTE detection and severity evaluation. However, their independent prognostic value requires confirmation in long-term follow-up studies.

## Supplementary information

**Additional file:** **Supplementary Table S1.** Comparison of the sub-sample and the total sample. **Supplementary Table S2.** Intra-observer ICC analysis. **Supplementary Table S3.** Inter-observer ICC analysis. **Supplementary Table S4.** Multivariable linear regression analysis of apical diastolic geometric parameters. **Supplementary Table S5.** Multivariable linear regression analysis of apical systolic geometric parameters

## Data Availability

The data used or analyzed during the current study are available from the corresponding author on reasonable request.

## Abbreviations

AUC: Area under the curve
CI: Cardiac index
CMR: Cardiac magnetic resonance imaging
CO: Cardiac output
CPTE: Chronic pulmonary thromboembolism
CTEPD: Chronic thromboembolic pulmonary disease
CTEPH: Chronic thromboembolic pulmonary hypertension
dPAP: Diastolic pulmonary arterial pressure
dRVP: Diastolic right ventricular pressure
ED: End-diastole
ES: End-systole
FD: Fractal dimension
ICC: Intraclass correlation coefficient
IDI: Integrated discrimination improvement
mPAP: Mean pulmonary arterial pressure
mRAP: Mean right atrial pressure
mRVP: Mean right ventricular pressure
NRI: Net reclassification improvement
PAR: Perimeter-area ratio
PAWP: Pulmonary artery wedge pressure
PVR: Pulmonary vascular resistance
PVRI: Pulmonary vascular resistance index
RHC: Right heart catheterization
ROC: Receiver operating characteristic
RV: Right ventricle
RVEF: Right ventricular ejection fraction
RVEDVI: Right ventricular end-diastolic volume index
sPAP: Systolic pulmonary arterial pressure
sRVP: Systolic right ventricular pressure
SvO₂: Mixed venous oxygen saturation
*V*/*Q*: Ventilation/perfusion
