# Supplementary material for: Geometric morphological parameters from cardiac magnetic resonance imaging in assessment of chronic pulmonary thromboembolism, a prospective pilot study
Source: Eur Radiol Exp. 2026 Sep 21;10:138. doi: 10.1186/s41747-026-00811-1 (PMC13594057; doi:10.1186/s41747-026-00811-1)
Supplement: Supplementary file 1 [file 41747_2026_811_MOESM1_ESM.docx]

**Supplementary Tables**

**Supplementary Table S1 . Comparison of sub-sample and total-sample**

| **Variables** | **Sub-sample (n=32)** | **Total-sample (n=106)** | ***p*** |
| --- | --- | --- | --- |
| Age (years old) | 52.34±12.92 | 53.09±12.54 | 0.769 |
| Gender (male/female) | 18/14 | 58/48 | 0.879 |
| Height (m) | 1.67±0.07 | 1.67±0.07 | 0.791 |
| Weight (kg) | 71.37±11.07 | 71.97±11.92 | 0.801 |
| BMI (kg/m^2^) | 25.33±3.40 | 25.59±3.26 | 0.701 |
| BSA (m^2^) | 1.80±0.16 | 1.81±0.17 | 0.969 |
| SBP (mmHg) | 120.15±16.14 | 123.90±16.18 | 0.252 |
| DBP (mmHg) | 78.75±11.76 | 82.33±13.25 | 0.172 |
|  | **CTEPH-sub-sample (n=11)** | **CTEPH-total-sample (n=37)** |  |
| Age (years old) | 55.00±14.57 | 56.65±14.49 | 0.742 |
| Gender (male/female) | 7/4 | 22/15 | 0.804 |
| Height (m) | 1.69±0.09 | 1.67±0.08 | 0.484 |
| Weight (kg) | 63.18±9.63 | 66.48±11.15 | 0.379 |
| BMI (kg/m^2^) | 22.00±2.72 | 23.70±3.19 | 0.118 |
| BSA (m^2^) | 1.72±0.16 | 1.74±0.17 | 0.709 |
| SBP (mmHg) | 108.36±15.12 | 113.91±15.72 | 0.305 |
| DBP (mmHg) | 68.81±10.02 | 74.21±13.26 | 0.220 |
|  | **CTEPD-sub-sample (n=5)** | **CTEPD-total-sample (n=16)** |  |
| Age (years old) | 46.40±21.46 | 45.56±17.05 | 0.929 |
| Gender (male/female) | 2/3 | 7/9 | 0.647 |
| Height (m) | 1.67±0.07 | 1.68±0.06 | 0.850 |
| Weight (kg) | 81.80±12.39 | 75.25±13.73 | 0.354 |
| BMI (kg/m^2^) | 29.02±1.83 | 26.48±3.51 | 0.141 |
| BSA (m^2^) | 1.94±0.16 | 1.85±0.18 | 0.336 |
| SBP (mmHg) | 119.20±5.06 | 124.12±12.70 | 0.415 |
| DBP (mmHg) | 76.00±4.30 | 80.31±10.20 | 0.376 |
|  | **Healthy-sub-sample (n=16)** | **Healthy-total-sample (n=53)** |  |
| Age (years old) | 52.38±7.98 | 52.89±7.86 | 0.821 |
| Gender (male/female) | 9/7 | 29/24 | 0.914 |
| Height (m) | 1.66±0.07 | 1.67±0.07 | 0.848 |
| Weight (kg) | 73.75±7.70 | 74.81±10.66 | 0.713 |
| BMI (kg/m^2^) | 26.47±2.01 | 26.64±2.64 | 0.811 |
| BSA (m^2^) | 1.82±0.12 | 1.84±0.15 | 0.684 |
| SBP (mmHg) | 128.56±14.20 | 130.81±13.83 | 0.573 |
| DBP (mmHg) | 86.43±8.74 | 88.60±10.71 | 0.464 |

**Supplementary Table S2. Intra‑observer ICC analysis**

| **Variables** | **Level** | **ICC (2，1)** | **95%CI** |
| --- | --- | --- | --- |
| RVFD | d1 | 0.920 | 0.829-0.962 |
|  | d2 | 0.947 | 0.891-0.974 |
|  | d3 | 0.918 | 0.839-0.959 |
|  | s1 | 0.882 | 0.773-0.941 |
|  | s2 | 0.927 | 0.857-0.964 |
|  | s3 | 0.907 | 0.818-0.953 |
| RVPAR | d1 | 0.964 | 0.928-0.982 |
|  | d2 | 0.955 | 0.910-0.978 |
|  | d3 | 0.945 | 0.891-0.973 |
|  | s1 | 0.919 | 0.829-0.961 |
|  | s2 | 0.921 | 0.844-0.961 |
|  | s3 | 0.955 | 0.911-0.978 |
| LVFD | d1 | 0.874 | 0.758-0.936 |
|  | d2 | 0.852 | 0.718-0.925 |
|  | d3 | 0.823 | 0.710-0.902 |
|  | s1 | 0.844 | 0.705-0.921 |
|  | s2 | 0.862 | 0.739-0.930 |
|  | s3 | 0.904 | 0.835-0.948 |
| LVPAR | d1 | 0.738 | 0.587-0.851 |
|  | d2 | 0.872 | 0.785-0.931 |
|  | d3 | 0.912 | 0.850-0.953 |
|  | s1 | 0.932 | 0.865-0.966 |
|  | s2 | 0.951 | 0.902-0.976 |
|  | s3 | 0.925 | 0.852-0.962 |

d1: end-diastolic basal level; d2: end-diastolic papillary muscle level; d3: end-diastolic apical level; s1: end-systolic basal level; s2: end-systolic papillary muscle level; s3: end-systolic apical level; RVFD, right ventricular fractal dimension; RVPAR, right ventricular perimeter-area ratio; LVFD, left ventricular fractal dimension; LVPAR, left ventricular perimeter-area ratio

**Supplementary Table S3. Inter‑observer ICC analysis**

| **Variables** | **Level** | **ICC (2，1)** | **95%CI** |
| --- | --- | --- | --- |
| RVFD | d1 | 0.876 | 0.710-0.944 |
|  | d2 | 0.910 | 0.825-0.955 |
|  | d3 | 0.916 | 0.759-0.965 |
|  | s1 | 0.765 | 0.565-0.879 |
|  | s2 | 0.868 | 0.729-0.935 |
|  | s3 | 0.897 | 0.747-0.954 |
| RVPAR | d1 | 0.929 | 0.627-0.976 |
|  | d2 | 0.908 | 0.610-0.967 |
|  | d3 | 0.945 | 0.855-0.976 |
|  | s1 | 0.827 | 0.522-0.928 |
|  | s2 | 0.932 | 0.648-0.977 |
|  | s3 | 0.899 | 0.756-0.954 |
| LVFD | d1 | 0.850 | 0.714-0.924 |
|  | d2 | 0.838 | 0.695-0.917 |
|  | d3 | 0.732 | 0.521-0.859 |
|  | s1 | 0.808 | 0.644-0.901 |
|  | s2 | 0.784 | 0.603-0.888 |
|  | s3 | 0.857 | 0.728-0.927 |
| LVPAR | d1 | 0.768 | 0.579-0.880 |
|  | d2 | 0.807 | 0.640-0.901 |
|  | d3 | 0.865 | 0.743-0.932 |
|  | s1 | 0.851 | 0.717-0.924 |
|  | s2 | 0.885 | 0.777-0.942 |
|  | s3 | 0.818 | 0.661-0.906 |

d1: end-diastolic basal level; d2: end-diastolic papillary muscle level; d3: end-diastolic apical level; s1: end-systolic basal level; s2: end-systolic papillary muscle level; s3: end-systolic apical level; RVFD, right ventricular fractal dimension; RVPAR, right ventricular perimeter-area ratio; LVFD, left ventricular fractal dimension; LVPAR, left ventricular perimeter-area ratio

**Supplementary Table S4. Multivariable linear regression analysis of apical diastolic geometric parameters**

| **Dependent variable** | **Independent variable** | **B (95% CI)** | **β** | ***p*-value** |
| --- | --- | --- | --- | --- |
| **PVR (Wood)** | Age (years old) | 0.131  (0.028, 0.247) | 0.346 | 0.010 |
|  | RVFD-d3 | 44.57  (-82.33, 135.28) | 0.109 | 0.408 |
|  | RVEF (%) | -0.250  (-0.392, -0.125) | -0.395 | 0.002 |
| **mPAP (mmHg)** | Age (years old) | 0.284  (0.066, 0.484) | 0.298 | 0.021 |
|  | RVFD-d3 | 149.70  (-110.98, 373.04) | 0.145 | 0.254 |
|  | RVEF (%) | -0.748  (-1.082, -0.489) | -0.469 | <0.001 |
| **PVR (Wood)** | Age (years old) | 0.144  (0.046, 0.233) | 0.381 | 0.002 |
|  | RVPAR-d3 | -22.23  (-51.16, 7.66) | -0.172 | 0.210 |
|  | RVEF (%) | -0.209  (-0.388, -0.083) | -0.330 | 0.019 |
| **mPAP (mmHg)** | Age (years old) | 0.330  (0.093, 0.514) | 0.346 | 0.004 |
|  | RVPAR-d3 | -68.02  (-158.08, -8.59)⁺⁺ | -0.208 | 0.040 |
|  | RVEF (%) | -0.626  (-0.959, -0.352) | -0.393 | <0.001 |

Note: Variance inflation factors (VIF) were <2 for all predictors, indicating no severe multicollinearity.
Abbreviations: FD: fractal dimension; PAR: perimeter-area ratio; RVEF: right ventricular ejection fraction; PVR: pulmonary vascular resistance; mPAP: mean pulmonary arterial pressure

**Supplementary Table S5. Multivariable linear regression analysis of apical systolic geometric parameters**

| **Dependent variable** | **Independent variable** | **B (95% CI)⁺** | **β** | ***p*-value** |
| --- | --- | --- | --- | --- |
| **PVR (Wood)** | Age (years old) | 0.139  (0.046, 0.234) | 0.367 | 0.008 |
|  | RVFD-s3 | 56.92  (-21.15, 134.45) | 0.143 | 0.133 |
|  | RVEF (%) | -0.218  (-0.366, -0.077) | -0.345 | 0.006 |
| **mPAP (mmHg)** | Age (years old) | 0.315  (0.094, 0.520) | 0.330 | 0.006 |
|  | RVFD-s3 | 166.35  (-67.96, 394.14) | 0.165 | 0.137 |
|  | RVEF (%) | -0.661  (-1.036, -0.331) | -0.415 | <0.001 |
| **PVR (Wood)** | Age (years old) | 0.138  (0.048, 0.222) | 0.366 | 0.005 |
|  | RVPAR-s3 | -26.64  (-54.38, -1.76) | -0.251 | 0.041 |
|  | RVEF (%) | -0.145  (-0.315, 0.019) | -0.229 | 0.089 |
| **mPAP (mmHg)** | Age (years old) | 0.311  (0.093, 0.493) | 0.326 | 0.002 |
|  | RVPAR-s3 | -84.64  (-159.09, 2.56) | -0.317 | 0.062 |
|  | RVEF (%) | -0.415  (-0.821, -0.018) | -0.261 | 0.040 |

Note: Variance inflation factors (VIF) were <2 for all predictors, indicating no severe multicollinearity.
Abbreviations: FD: fractal dimension; PAR: perimeter-area ratio; RVEF: right ventricular ejection fraction; PVR: pulmonary vascular resistance; mPAP: mean pulmonary arterial pressure
